# Surgical Management of Uterine Fibroids at Aminu Kano Teaching Hospital

**DOI:** 10.1155/2012/702325

**Published:** 2011-11-10

**Authors:** Abiodun Omole-Ohonsi, Francis Belga

**Affiliations:** ^1^Department of Obstetrics and Gynaecology, Bayero University, Kano and Aminu Kano Teaching Hospital, PMB 3452, Kano 700282, Nigeria; ^2^Department of Obstetrics and Gynecology, Murtala Mohammed Specialist Hospital, Kano, Nigeria

## Abstract

*Objective*. To determine the influence of age and parity on the surgical management of uterine fibroids, clinical presentation, presence of pelvic adhesions, cadre of surgeons, and postoperative complications at the Aminu Kano Teaching Hospital, Kano, Nigeria. *Methods*. A retrospective analysis of 105 cases of uterine fibroids that were managed between 1st January 2003 and 31st December 2007. *Results*. The period prevalence of uterine fibroids was 24.7% of all major gynecological operations. The mean age was 35.8 ± 7.6 and mean parity 4.7 ± 2.8. Abdominal hysterectomy accounted for 58.1% of the cases and myomectomy 41.9%. The odd of using abdominal hysterectomy was about twice that of myomectomy. Pelvic adhesions were found in 67.6% of the cases. Menorrhagia (86.7%) was the commonest symptom, while post operative anemia and pyrexia showed significant association with myomectomy. There was no maternal mortality. *Conclusion*. Surgical operations for uterine fibroids are safe and common kind of gynecological operations at the Aminu Kano Teaching Hospital. Uterine fibroid is associated more with high parity and dominance of abdominal hysterectomy over myomectomy, because early girl marriage is common in our community.

## 1. Introduction

Uterine fibroids are the most common pelvic tumour and the most common noncancerous tumors in women of childbearing age [1]. As many as 1 in 5 women may have fibroids during their childbearing years, and it usually affect women over the age of 30 years [1, 2]. It is estimated that 20 to 30% of women above the age of 30 years harbor uterine fibroids, which account for 3.2–7.6% of new gynaecological cases and 68.1% of hysterectomies [3–5].

An incidence of 17.9%–26% has been found at laparoscopy in some Nigerian studies [6, 7], which is much higher than 11% reported from Europe and the USA [8]. Overexpression of estrogen receptor (ER) alpha genotype and aromatase, which has been found to correlate with incidence and size of uterine fibroids, is particularly pronounced in Afro-American women [9, 10] and may explain why African American women are at three to five times greater risk for fibroids than white women and Negros 3–9 times than Caucasians [10]. Aromatase inhibitors are currently being considered for prevention and treatment of uterine fibroids [11]. 

The cause of uterine fibroids is not known, but there are several risk factors [1, 2]. Known risk factors are Afro-American descent, nulliparity, obesity, polycystic ovary syndrome, diabetes, and hypertension [12, 13]. Current working hypothesis is that gene predisposition, prenatal hormone exposure, and effects of hormones, growth factors, and xenoestrogens cause fibroid growth [12]. 

Genetic and hereditary causes are being considered [14, 15]. First degree relatives have 2–5-fold risk, and nearly 6- fold risk when considering only early onset cases [14]. A positive family history suggests genetic factors, and a gene encoding for fibroid development has been suggested [15]. 

Fibroid growth is strongly dependent on estrogen and progesterone, which are regarded as growth promoting [16, 17]. Paradoxically, fibroids will rarely grow during pregnancy despite very high steroid hormone levels, and pregnancy appears to exact a protective effect. The mechanism(s) by which pregnancy exerts its protective effects is unclear, but may be mediated by an interaction of estrogen, prostaglandins, and oxytocin [18, 19]. This is said to explain why the incidence of uterine fibroids is decreased with increasing number of term pregnancies [18], although studies from Nigeria [5] did not agree with this.

Uterine fibroids, although asymptomatic in many women, are often detected in women undergoing infertility evaluation in many black communities [6, 7]. They tend to give symptoms from the age of 30 years to the end of the reproductive life [12, 13]. They grow very slowly, but their rate of growth varies from patient to patient or under differing circumstances, with 90% of them ceasing to grow or even regressing after the menopause [2], probably because the ER-beta, ER-alpha, and progesterone receptors are overexpressed in premenopausal fibroids [14, 20–23]. 

Hysterectomy which is a major procedure that removes the uterus is the definitive treatment for uterine fibroid [2, 9, 24]. Myomectomy which removes only the fibroids and leaves the healthy areas of the uterus in place is usually reserved for women under the age of 40 years, who are of low parity and desire to maintain their fertility, when the procedure is surgically feasible and there is a reasonably good chance of subsequent pregnancy [9, 25–27]. The rates of hysterectomy for fibroids vary between 32% and 70.4% [4, 5, 9] and myomectomy between 15.8% and 86% [3–5].

Newer techniques include the use of laser vaporization, ultrasonic diathermy coagulation to burn the fibroid nodules, and laparoscopic myomectomy for abdominal myomectomy [2]. The use of gonadotrophic releasing hormone analogs and uterine artery embolization (UAE), which reduce the blood supply to the uterus and fibroids, making them shrink [1, 2], are procedures that would be extremely popular in developing countries, where the culture often makes women resent undergoing major surgery or losing their uterus [9]. However, the expensive equipments and cost of the procedures make them often out of the reach of most developing countries [9]. 

It is evident that the determining factors in the management of uterine fibroids and the outcome of treatment vary widely in different communities. It is therefore the purpose of this study to determine the factors that influence the management of uterine fibroids in our community, the presentation and outcome.

## 2. Material and Methods

A retrospective analysis of 105 cases of uterine fibroids that were managed at the Aminu Kano Teaching Hospital, Kano, Nigeria, between the 1st January 2003 and 31st December 2007. The patients' identification data were retrieved from the gynecological ward admission and discharge record books and theatre's operation register. Their case notes were retrieved from the Medical Records Department and analyzed for incidence, age, parity, clinical presentation, presence of pelvic adhesions, type of surgical treatment, and postoperative complications.

During the study period, myomectomy was done using the tourniquet method, in which a tourniquet was applied around the lower uterine segment and below the fibroids, to achieve mechanical vasoconstriction on the ascending uterine artery bilaterally [28, 29]. A tourniquet time was kept, and the tourniquet was released after 30 minutes and reapplied after 5 minutes to reestablish blood flow and prevent irreversible damage to the uterine muscle cells [28, 29]. 

Postoperatively, a packed cell volume of less than 30% was considered as anemia, a temperature of 38°C or more on two consecutive days after the first post operative day was considered as pyrexia, dysuria and/or frequency of micturtion with positive urine microbiology culture was taken as urinary tract infection (UTI), and local erythema or suppuration was considered as wound infection. 

The data obtained were recorded using tables. Statistical analysis was done with Chi-square test using a commercial statistical package (SPSS/PC version 11.0, SPSS Inc., Chicago, Ill, USA). 

The odds ratio (OR) and 95% confidence interval (CI) were determined where appropriate. A *P* value of less than 0.05 was considered significant.

## 3. Results

During the study period, surgical operations for uterine fibroids were carried out in 115 cases out of 465 major gynecological operations that were performed, giving a period prevalence of 24.7% of major gynecological operations for uterine fibroids. Only 105 case notes were retrieved from the Medical Records Department giving a retrieval rate of 91.3%. Abdominal hysterectomy was performed in 58.1% of the cases, while 41.9% had abdominal myomectomy, the odd of using abdominal hysterectomy was about twice that of myomectomy (OR = 1.92. CI = 1.07–3.46, *P* < 0.05). There was no case of vaginal hysterectomy or endoscopic surgery, and all the hysterectomies were total abdominal hysterectomy. Pelvic adhesions were found in 67.6% of the cases, while 32.4% had clean pelvic cavity. All the surgeries were done with consultant gynecologists participating.

The age range among the patients was 26–55 years, with a mean age of 35.8 ± 7.6. The highest frequency (65.7%) was in the 30–34-year age group, while the least (6.7%) was among the 25–29-year age group. The odd of having hysterectomy was highest among 35–39-year age group (OR = 5.72, CI = 1.43–26.52, *P* < 0.05), while there was no statistically significant difference in the odd of having hysterectomy or myomectomy among the 30–34-year age group (OR = 0.40, CI = 0.15–1.02, *P* > 0.05). All the patients in the 20–29-year age group had myomectomy, while the only nulliparous patients among the 40-year-or-more age group had hysterectomy Table 1.

The parity range was from 0 to 12, with a mean parity of 4.7 ± 2.8. Among the patients, 6.7% were nulliparous, while 93.3% were of parous, with at least one living child. Among them, 33 women (31.4%) were grand multiparae, which accounted for the highest frequency among the patients, while the least frequency was among the nulliparae. The use of hysterectomy was significantly higher among Para 4 and Para ≥5, while myomectomy was significantly higher among Para 0–2. There was no significant difference in the use of hysterectomy or myomectomy among Para 3. Two nulliparous women in the 30–34-year age group had hysterectomy, because of huge fibroid and technical difficulties that were encountered during myomectomy.  Table 2.

Menorrhagia (86.7%) was the commonest symptom, followed by abdominal swelling (61.0%), lower abdominal pain (55.2%), and dysmenorrhoea (38.1%). Infertility (23.8%) accounted for the least frequency, with majority of them (80.0%) having secondary infertility Table 3. 

Postoperative anemia (41.0%) was the most common complication, followed by post operative pyrexia (33.3%), UTI (8.6%), and wound infection (15.2%). Postoperative anemia (OR = 5.37, CI = 2.13–13.77, *P* < 0.05), and pyrexia (OR = 4.47, CI = 1.74–11.70, *P* < 0.05), showed statistically significant association with myomectomy. Postoperative anemia occurred 5 times more, while postoperative pyrexia occurred 4 times more among patients who had myomectomy compared to hysterectomy. There was no statistically significant difference (*P* > 0.05) in the frequency of urinary tract infection (UTI) and wound infection in the two groups Table 4. There was no maternal mortality.

## 4. Discussion

The period prevalence of 24.7% of major gynaecological operations for uterine fibroids in this study is similar to the findings in other studies from Nigeria [27], but lower than reports from Europe [8], probably because uterine fibroid is more common among the black race [9]. 

In this study, uterine fibroids occurred most often in the third decade of life, which agrees with the findings of other studies [27, 30, 31], which may probably be because uterine fibroids is uncommon before the age of 30 years and after menopause [9]. 

Majority of the patients were of high parity because of early girl marriage and childbearing in our community, and uterine fibroid was associated more with secondary infertility in this study, which does not agree with the findings in the study from Ilorin [27] in north-Central Nigeria, Enugu [31], and Abakaliki [32] in-south-eastern Nigeria, and Addis Ababa in Ethiopia [30], where women delay marriage, and uterine fibroid is associated more with low parity and primary infertility [27, 32]. This may be because prolonged periods of voluntary infertility from delayed age of marriage are usually associated with development of uterine fibroids and primary infertility [1, 2]. This may also explain why the overall hysterectomy rate was twice as much as that of myomectomy in this study, while myomectomy was used more than hysterectomy in the study from Ilorin [27], Enugu [31] and Abakaliki [32] in Nigeria and Addis Ababa in Ethiopia [30]. A Study from Gombe [33] in-north-eastern Nigeria, which is also predominantly Islamic communities like ours, where early girl marriage is common, recorded low frequency of nulliparity among their hysterectomy patients, majority of whom had uterine fibroids, compared to similar study from Ibadan [34] in-south-west Nigeria where women delay marriage, in which uterine fibroids was also the commonest indication. 

Majority of the women had pelvic adhesions, which agree with other studies from Nigeria [7, 26, 27]. The high association of uterine fibroids with pelvic adhesions has been attributed to the high prevalence of pelvic inflammatory disease (PID), previous caesarean section, and laparotomy in developing countries [26, 33], which may cause tubal disease and contribute to the significant association with infertility [1, 2]. Early girl marriage and childbearing in our community before the age when uterine fibroids are common may explain why menorrhagia was the commonest clinical presentation and infertility the least in this study, while infertility (mainly primary) was the commonest clinical presentation in the study from Ilorin [27] and Abakaliki [32] in Nigeria, where women delay marriage and childbearing.

The high association of uterine fibroids with pelvic inflammatory disease and pelvic adhesions, and the large size of most fibroids in developing countries [9] has been found to be the reason why vaginal hysterectomy is not commonly employed in the management of uterine fibroids, because of the technical difficulties that may be involved [9]. This may explain why vaginal hysterectomy was not employed in the management of uterine fibroids in this study. With the advent of laparoscopic-assisted vaginal hysterectomy in our hospital, smaller fibroids may be removed per vaginam. 

There was no case of hysterectomy carried out among women who were less than 30 years, probably because hysterectomy among women in that age group is not done for emotional reasons [9]. Two nulliparous women had hysterectomy, because of huge uterine fibroids and technical difficulties at surgery. Huge uterine fibroid as a result of delay in presentation is a common occurrence in developing countries like Nigeria and has been reported to be a cause of emergency hysterectomy as a result of technical difficulties encountered during myomectomy [34], which further emphasizes the need to obtain consent for hysterectomy in addition, before embarking on myomectomy.

 Most of the women aged ≥40 years had hysterectomy, because they were parous, and also because of the low probability of further pregnancies at that age compared to future complications [9], which advised in favour of hysterectomy, in order to give them good quality of life. The only case of myomectomy among the 40-year-and-above age group, was the only case of primary infertility in that group, so as to enable her to benefit from in vitro fertilization and embryo transfer, which she can afford and agreed to during preoperative counseling. Myomectomy and recourse to IVF-ET is a possibility that is currently being explored in the management of uterine fibroids in older women with primary infertility [27]. 

Postoperative anaemia and pyrexia were the commonest postoperative morbidity, which agrees with other studies [1, 2]. Postoperative anaemia and pyrexia occurred more with myomectomy than hysterectomy, which may probably be due to bleeding into the fibroid cavities and peritoneum, with resultant reactionary pyrexia following myomectomy [2], so effort must be made to obliterate all dead spaces at surgery [1, 2]. 

The higher frequency of complications with myomectomy compared to hysterectomy in this study agree with other studies [2]. This can be reduced by using endoscopic methods like da Vinci Myomectomy, which is a new category of minimally invasive myomectomy, and the latest evolution in robotics technology, which combines the best of open and laparoscopic surgery [35]. With minimally invasive myomectomy using endoscopic methods, surgeons can remove uterine fibroids through small incisions with unmatched precision and control, and carry out comprehensive reconstruction of the uterine wall, regardless of the size or location of the fibroids. Among the potential benefits of minimally invasive myomectomy using endoscopic methods as compared to traditional open abdominal surgery are better opportunity for future pregnancy, significantly less pain, less blood loss, fewer complications with less scarring and possibility of uterine rupture during future pregnancies, a shorter hospital stay, and a faster return to normal daily activities [35]. We advocate the introduction of minimally invasive myomectomy using endoscopic methods in health facilities that perform myomectomy.

Wound infection and urinary tract infection which did not show statistically significant difference between myomectomy and hysterectomy cases may be a result of poor environmental and personal hygiene in our community in a developing country, and urethral catheterization, which has been known to predispose postoperative patients to urinary tract infection [9].

The uterine tourniquet which was applied round the lower uterine segment and below the fibroids during myomectomy in this study, is a mechanical vaso-occlusive technique to achieve mechanical vasoconstriction on the ascending uterine artery bilaterally [28, 29]. It has been found to be associated with low risk of haemorrhage and difficulty with securing haemostasis, as well as postoperative morbidity, shorter mean duration of operation and hospital stay [28, 29]. This may explain why only two cases of myomectomy were abandoned for hysterectomy, because of difficulty during the operation in this study.

There was no maternal mortality, probably because of meticulous care and the surgeries were done with consultant gynecologists participating. This calls for consultant's participation in surgeries for uterine fibroids.

## 5. Conclusion and Recommendations

Surgical operations for uterine fibroids are common gynaecological operations at the Aminu Kano Teaching Hospital. Myomectomy and hysterectomy which are the only modalities of management of uterine fibroids that are presently available are safe and effective, the choice of which should be individualized among the patients. Communities where early girl marriage and childbearing is practiced should expect uterine fibroids to be associated more with high parity, and dominance of abdominal hysterectomy over myomectomy. 

Introduction of minimally invasive myomectomy using endoscopic methods may reduce the higher frequency of complications that are associated with myomectomy, and laparoscopic-assisted vaginal hysterectomy may make smaller fibroids to be removed per vaginam.

## Figures and Tables

**Table 1:** Age and type of operation performed.

Age (years)	Hysterectomy	Myomectomy	OR	CI	*P* Value
25–29	—	6	—	—	Significant**
25–29	35	34	0.40	0.15–1.02	>0.05 (NS)
35–39	17	3	5.80	1.32–24.59	<0.05*
≥40	9	1	7.44	0.90–163.00	<0.05*

**Total**	**61 (58.1)**	**44 (41.9)**	**1.92**	**1.07–3.46**	<0.05*

*Statistically significant for hysterectomy

**Statistically significant for myomectomy

NS: Not statistically significant.

**Table 2:** Parity and type of operation performed on the patient.

Parity	Hysterectomy	Myomectomy	OR	CI	*P* value
O	2	5	0.26	0.03–1.65	<0.05**
1	3	8	0.23	0.05–1.06	<0.05**
2	3	10	0.18	0.04– 0.76	<0.05**
3	12	18	0.35	0.13–0.92	< 0.05**
4	13	2	5.69	1.12–38.84	<0.05*
≥5	28	1	36.48	4.85–757.27	<0.05*

**Total**	** 61 (58.1)**	** 44 (41.9)**	**1.92**	** 1.07–3.46**	<0.05*

*Statistically significant for hysterectomy

**Statistically significant for myomectomy.

**Table 3:** Clinical presentation of patients.

Presentation	Frequency *n* (%)
(i) Menstrual abnormalities	91 (86.7)
(ii) Abdominal swelling	64 (61.0)
(iii) Lower abdominal pain	58 (55.2)
(iv) Dysmenorrhoea	40 (38.1)
(v) Infertility	25 (23.8)
Primary	5 (20.0)
Secondary	20 (80.0)

**Table 4:** Postoperative Complications.

Complication	Frequency *n* (%)				
	Myomectomy	Hysterectomy	OR	CI	*P*-value
	*n* = 44	*n* = 61			
Anaemia	28	15	5.37	2.13–13.77	<0.05*
Fever	23	12	4.47	1.74–11.70	<0.05*
UTI	4	5	1.12	0.23–5.22	>0.05
Wound infection	9	7	1.98	0.60–6.61	>0.05

*Statistically significant.
